# Prediction of fat-free mass in young children using bioelectrical impedance spectroscopy

**DOI:** 10.1038/s41430-023-01317-4

**Published:** 2023-07-31

**Authors:** Jaz Lyons-Reid, Leigh C. Ward, José G. B. Derraik, Mya Thway-Tint, Cathriona R. Monnard, J. Manuel Ramos Nieves, Benjamin B. Albert, Timothy Kenealy, Keith M. Godfrey, Shiao-Yng Chan, Wayne S. Cutfield

**Affiliations:** 1 Liggins Institute, University of Auckland, Auckland, New Zealand; 2 School of Chemistry and Molecular Biosciences, University of Queensland, Brisbane, QLD Australia; 3 Department of Paediatrics: Child and Youth Health, University of Auckland, Auckland, New Zealand; 4 Environmental-Occupational Health Sciences and Non-communicable Diseases Research Group, Research Institute for Health Sciences, Chiang Mai University, Chiang Mai, Thailand; 5 Department of Women’s and Children’s Health, Uppsala University, Uppsala, Sweden; 6 Singapore Institute for Clinical Sciences, Agency for Science, Technology and Research (A*STAR), Singapore, Singapore; 7 Human Potential Translational Research Programme, Yong Loo Lin School of Medicine, National University of Singapore, Singapore, Singapore; 8 Nestlé Institute of Health Sciences, Nestlé Research, Société des Produits Nestlé S.A., Lausanne, Switzerland; 9 Department of Medicine and Department of General Practice and Primary Health Care, University of Auckland, Auckland, New Zealand; 10 MRC Lifecourse Epidemiology Centre, University of Southampton, Southampton, UK; 11 NIHR Southampton Biomedical Research Centre, University of Southampton and University Hospital Southampton NHS Foundation Trust, Southampton, UK; 12 Department of Obstetrics & Gynaecology, National University of Singapore, Singapore, Singapore; 13 A Better Start – National Science Challenge, University of Auckland, Auckland, New Zealand

**Keywords:** Paediatrics, Obesity

## Abstract

**Background:**

Bioimpedance devices are practical for measuring body composition in preschool children, but their application is limited by the lack of validated equations.

**Objectives:**

To develop and validate fat-free mass (FFM) bioimpedance prediction equations among New Zealand 3.5-year olds, with dual-energy X-ray absorptiometry (DXA) as the reference method.

**Methods:**

Bioelectrical impedance spectroscopy (SFB7, ImpediMed) and DXA (iDXA, GE Lunar) measurements were conducted on 65 children. An equation incorporating weight, sex, ethnicity, and impedance was developed and validated. Performance was compared with published equations and mixture theory prediction.

**Results:**

The equation developed in ~70% (*n* = 45) of the population (FFM [kg] = 1.39 + 0.30 weight [kg] + 0.39 length^2^/resistance at 50 kHz [cm^2^/Ω] + 0.30 sex [M = 1/F = 0] + 0.28 ethnicity [1 = Asian/0 = non-Asian]) explained 88% of the variance in FFM and predicted FFM with a root mean squared error of 0.39 kg (3.4% of mean FFM). When internally validated (*n* = 20), bias was small (40 g, 0.3% of mean FFM), with limits of agreement (LOA) ±7.6% of mean FFM (95% LOA: –0.82, 0.90 kg). Published equations evaluated had similar LOA, but with marked bias (>12.5% of mean FFM) when validated in our cohort, likely due to DXA differences. Of mixture theory methods assessed, the SFB7 inbuilt equation with personalized body geometry values performed best. However, bias and LOA were larger than with the empirical equations (–0.43 kg [95% LOA: –1.65, 0.79], *p* < 0.001).

**Conclusions:**

We developed and validated a bioimpedance equation that can accurately predict FFM. Further external validation of the equation is required.

## Introduction

There is increasing evidence that body composition in early life is related to later health outcomes [1–8]. However, as early childhood is characterized by rapid growth and changes to body composition, gaining an understanding of the changes that occur to fat and fat-free masses (FM and FFM) can be challenging [9]. Several longitudinal studies have described the evolution of body composition throughout infancy, but there are limited data describing changes from 2 to 5 years [10, 11].

At this age, few tools are capable of measuring body composition and most are unsuitable for field use. Although quantitative magnetic resonance (i.e., EchoMRI) is suitable for use across the age span, it is highly specialized and costly equipment, available at few research centers globally [12]. Other techniques, such as air displacement plethysmography and dual-energy X-ray absorptiometry (DXA), are more widely available but are impacted by movement [13, 14]. At this age, compliance can be problematic. There is a need for an easy-to-use technique with a short measurement time. Bioelectrical impedance analysis (BIA), which involves measurement of the opposition to a small alternating electrical current as it passes through the body, offers promise as a useful field tool [15]. However, the technique relies on the availability of a prediction equation appropriate for the population being studied. Bioelectrical impedance spectroscopy (BIS), which measures impedance across a range of frequencies, can determine body composition without prediction equations by fitting measured impedances to a Cole model of resistance versus reactance to estimate resistance at zero (i.e., very low) and infinite (i.e., very high) frequencies. Body water volumes are predicted using a biophysical model-based approach (i.e., mixture theory) which accounts for the non-conducting compartments of the human body (i.e., cells) [16]. Nonetheless, population-specific coefficients are required for this approach.

Few bioimpedance equations exist for use in early childhood (2–5 years) [17–24]. Rush et al. [18] developed prediction equations for FFM among a multi-ethnic cohort of New Zealand 2-year olds (*n* = 77) using DXA as a reference standard, but they did not validate their equations. Ejlerskov et al. [17] developed and validated prediction equations for FFM among 3-year olds (*n* = 99); however, these equations were developed among Danish children and may not be applicable to other ethnicities. Other equations developed for use in early childhood include those developed among wide age ranges [19–22] and those developed among homogeneous Asian cohorts [23, 24]. A procedure for adjustment of BIS coefficients has also been derived for use among children aged over 4 years [25].

We aimed to evaluate bioimpedance in early childhood by developing prediction equations for FFM based on DXA among New Zealand preschool children. These equations were subsequently compared to the previously published equations by Ejlerskov et al. [17] and Rush et al. [18]. Furthermore, we evaluated whether FFM could be accurately estimated with BIS using mixture theory prediction.

## Methods

Participants were healthy children born between April 2016 and January 2019 to New Zealand mothers participating in the Nutritional Intervention Preconception and During Pregnancy to Maintain Healthy Glucose Metabolism and Offspring Health (NiPPeR) study [26]. Procedures involving human participants were approved by the Northern A Health and Disability Ethics Committee New Zealand (15/NTA/21/). Written informed consent was obtained from the mothers of the participants. The NiPPeR trial was registered on 16 July 2015 (ClinicalTrials.gov NCT02509988; Universal Trial Number U1111-1171-8056).

Comprehensive inclusion criteria for the NiPPeR study are reported in Supplementary Table S1. Only children without congenital anomalies that may impact body composition were included in this study, who had weight, height, and valid BIS and DXA data collected on the same occasion at 3.5 years (*n* = 65).

### Anthropometry

Standing height was measured in triplicate to the nearest 0.1 cm using a calibrated SECA 213 portable stadiometer (SECA, Hamburg, Germany). Weight was obtained while lightly clothed using calibrated SECA 899 scales and was measured to the nearest 100 g.

### Dual-energy X-ray absorptiometry

Children were measured by trained research staff according to a standardized procedure on a GE Lunar iDXA (enCORE v17, pediatric mode) as detailed previously [27]. Briefly, median height and weight were entered into the calibrated DXA machine to inform scan mode selection and the length of the area to be scanned. The coefficient of variation from daily block phantom calibrations over the duration of the study was 0.23% for bone mineral density. Children were measured in light clothing, without metal, lying supine on the measurement bed. Scans with movement artifact were graded, with scans affected by considerable movement artifact excluded from analyses. Else, limb reflection was used when there was missing or duplication in either the left or right arm/leg [28]. All body composition values are reported as whole-body estimates.

### Bioelectrical impedance spectroscopy

BIS measurements were obtained using the ImpediMed SFB7 (ImpediMed, Brisbane, Australia) as described previously [29]. Briefly, electrodes were used to attach sense leads to the dorsum of the wrist and ankle, and source leads to the palm at the metacarpal heads and the sole at the metatarsal heads on the same side of the body. Most children (90%) were measured on the left side of the body. There were no differences in mean impedance parameters between children measured on the left versus the right (*n* = 58 vs 7; all *p* > 0.9).

Children were measured on an examination bed with legs apart and arms separated from the torso at a 30–45° angle. The protocol required children to be supine for 4 min prior to measurement. In lieu of requiring the child to fast and void their bladder prior to measurement, which would not have been feasible, the time of last meal and last bladder void were also recorded. Measurements were made in triplicate using the continuous setting of the device (coefficient of variation for resistance at 50 kHz, R_50_ = 0.17%). Cole plots were examined to ensure data quality, and measurements were repeated if movement occurred or if the Cole plots were poorly fitted [30].

We considered multiple parameters for inclusion in the equations (R_50_; resistance at zero kHz, R_0_; resistance at infinite kHz, R_∞_; and impedance at the characteristic frequency, Z_c_); however, predictive ability was comparable. Therefore, we used R_50_ as most single-frequency BIA devices use this frequency. This parameter was also used in the previously published equations [17, 18]:

$${{{\mathrm{FFM}}}}_{{{{\mathrm{Ejlerskov}}}}}\left( {{{\mathrm{g}}}} \right) = - 2784.4 + 327.2{{{\mathrm{L}}}}^2/{{{\mathrm{R}}}}_{50} + 223.8{{{\mathrm{Wt}}}} + 76.8{{{\mathrm{Ht}}}} + 417.6{{{\mathrm{S}}}}$$

$${{{\mathrm{FFM}}}}_{{{{\mathrm{Rush}}}}}\left( {{{{\mathrm{kg}}}}} \right) = - 2.490 + 0.367{{{\mathrm{L}}}}^2/{{{\mathrm{R}}}}_{50} + 0.188{{{\mathrm{Wt}}}} + 0.077{{{\mathrm{Ht}}}} + 0.273{{{\mathrm{S}}}}$$

Abbreviations: L^2^/R_50_, impedance index at 50 kHz (cm^2^/Ω); Wt, weight (kg); Ht, standing height (cm); S, sex (M = 1/F = 0).

BIS was also evaluated using mixture theory prediction. We evaluated multiple approaches from the literature, including the default SFB7 adult coefficients, the Moissl method [31], the original Xitron 4000B method [25], and the Xitron Hydra method [16]. Additionally, we evaluated the SFB7 method using personalized body geometry (Kb) values, instead of the default value (Kb = 4.3) [32]. FFM was then estimated from total body water (TBW) by dividing TBW by age- and sex-specific hydration factors [10].

### Data analyses

BIS prediction equations were developed in a manner similar to that used among our cohort at 6 weeks and 6 months [33]. Children with valid data were split into derivation (~70%) and validation (~30%) cohorts using a random number generator stratifying by sex. Predictive regression equations were developed using bi-directional stepwise multiple linear regression analysis. Differences between the derivation and validation cohorts were assessed using two-sample *t-*tests for continuous variables and Fisher’s exact tests for categorical variables. Assumptions of multiple linear regression were checked using scatterplots, correlation matrixes, variance inflation factors, plots of standardized residuals against predicted values, and Q-Q plots.

The equations were developed to predict FFM, using either simple anthropometric equations (based on height, L [cm]) or equations based on impedance (as the impedance index, L^2^/R [cm^2^/Ω]). In addition to height/impedance index, weight, and sex, gestational age, birthweight *z* score [34], ethnicity, time since last meal (<30 min, 30 min–1 h, 1–2 h, >2 h), and time since last bladder void (<15 min, 15–30 min, 30 min–1.5 h, >1.5 h) were assessed. However, with the exception of ethnicity (self-reported maternal ethnicity—White Caucasian/Chinese/Indian/Other–collapsed into Asian/non-Asian), the inclusion of these parameters did not improve the predictive ability of the equations; therefore, they were disregarded.

Scale weights (Wt_scale_) were higher than DXA estimates of weight (+28 g [95% CI: 0.23, 0.32], *p* < 0.001). Therefore, to enable the estimation of FM, we also developed prediction equations for adjusted weight (Wt_adj_) using simple linear regression [17]. FM was calculated as follows:

$${{{\mathrm{Wt}}}}_{{{{\mathrm{adj}}}}} = 0.41 + 0.99{{{\mathrm{Wt}}}}_{{{{\mathrm{scale}}}}}$$

$${{{\mathrm{FM}}}} = {{{\mathrm{Wt}}}}_{{{{\mathrm{adj}}}}}-{{{\mathrm{FFM}}}}_{{{{\mathrm{pred}}}}}$$

The final anthropometry-based and impedance-based prediction equations were applied to the validation cohort, with agreement between estimated and reference body composition being assessed using mean absolute percentage error (MAPE), Passing and Bablok regression scatterplots [35], Pearson’s correlation coefficient (*r*), Lin’s concordance coefficient (CCC) [36], and two one-sided tests of equivalence [37]. Bland–Altman plots were used to assess intra-individual differences [38]. Estimates of body composition from the published equations by Ejlerskov et al. [17] and Rush et al. [18], as well as mixture theory prediction estimates, were validated among the entire cohort using the methods above.

Descriptive statistics are presented as means ± SD for continuous variables and *n* (%) for categorical variables. All statistical analyses were conducted in R (version 4.3.0, R Foundation for Statistical Computing, Vienna, Austria). Statistical significance was defined as *p* values <0.05.

## Results

### Study population

Complete data were available from 65 children (Supplementary Fig. S1): characteristics are detailed in Table 1. There were no differences between the development (*n* = 45) and validation (*n* = 20) cohorts (Supplementary Table S2).

**Table 1:** Characteristics of the study population.

	Males	Females
*n* (%)	25 (38.5%)	40 (61.5%)
Gestational age at birth (weeks)	39.4 ± 1.8	39.6 ± 1.4
Pre-term^a^	2 (8.0%)	2 (5.0%)
Term	23 (92.0%)	38 (95.0%)
Birthweight z-score^b^	0.34 ± 1.07	0.32 ± 0.99
Age at visit (days)	1244 ± 81	1225 ± 52
Scale weight (kg)	15.7 ± 2.0	15.3 ± 1.7
Weight z-score^c^	0.28 ± 0.96	0.34 ± 0.84
Standing height (cm)	100.3 ± 3.4	98.6 ± 3.5
Height z-score^c^	0.40 ± 0.78	0.26 ± 0.95
BMI (kg/m^2^)	15.5 ± 1.2	15.7 ± 1.0
BMI z-score^c^	0.00 ± 0.97	0.26 ± 0.73
Fat-free mass^d^ (kg)	12.0 ± 1.4	11.1 ± 1.0
Fat mass^d^ (kg)	4.0 ± 0.9	4.5 ± 0.9
Fat mass^d^ (%)	25.1 ± 3.5	28.6 ± 3.5
Lean mass^d^ (kg)	11.4 ± 1.3	10.6 ± 1.0
Bone mineral content^d^ (g)	545 ± 65	517 ± 56
Resistance at 0 kHz (Ω)	786 ± 67	826 ± 76
Resistance at ∞ kHz (Ω)	597 ± 60	627 ± 64
Impedance at Fc^e^ (Ω)	694 ± 63	730 ± 69
Resistance at 50 kHz (Ω)	721 ± 65	757 ± 70
Ethnicity		
White Caucasian	16 (64.0%)	30 (75.0%)
Chinese	4 (16.0%)	5 (12.5%)
South Asian	3 (12.0%)	2 (5.0%)
Other	2 (8.0%)	3 (7.5%)
Randomization group		
Intervention	12 (48.0%)	20 (50.0%)
Control	13 (52.0%)	20 (50.0%)

Data are means ± SD for continuous variables and *n* (%) for categorical variables.

^a^Average gestational age 35.7 weeks (range: 35.0–36.4).

^b^INTERGROWTH-21st birthweight z-score.

^c^World Health Organization age- and sex-standardized z-score.

^d^Whole-body estimates from DXA.

^e^Impedance at the characteristic frequency (Fc).

### Prediction of fat-free mass

Table 2 outlines the developed prediction equations for FFM and associated model performance. Weight alone explained 73% of the variance in FFM; however, the root mean squared error (RMSE) was large at 0.61 kg, equivalent to 5.3% of mean FFM. The addition of length increased the proportion of explained variance to 79% and reduced the error to 0.53 kg. The substitution of length with the impedance index further increased explained variance to 86% and decreased the error to 0.42 kg (3.7% of mean FFM). The final equations, which additionally contained sex and ethnicity, explained 82 and 88% of the variation in FFM with errors of 0.47 and 0.39 kg (4.1 and 3.4% of mean FFM) for the anthropometry and impedance equations, respectively.

**Table 2:** Multivariable linear regression analysis of weight (W), sex (S), and ethnicity (E) in combination with height (L) or the impedance index (L^2^/R_50_) for predicting dual-energy X-ray absorptiometry fat-free mass (FFM) among the 3.5-year-old derivation cohort.

	aR^2^	RMSE	Standardized coefficients				Prediction equation for FFM
			W	L or L^2^/R_50_	S	E	
*All (n* *=* *45)*							
W	0.726	0.609 (5.3%)	0.856***				2.88 + 0.55W
W + L	0.788	0.529 (4.6%)	0.501***	0.438***			–9.19 + 0.32W + 0.16L
W + L + S	0.822	0.479 (4.2%)	0.557***	0.367***	0.195**		–7.41 + 0.36W + 0.13L + 0.47S
W + L^2^/R_50_	0.864	0.423 (3.7%)	0.406***	0.584***			1.49 + 0.26W + 0.44L^2^/R_50_
W + L^2^/R_50_ + S	0.875	0.402 (3.5%)	0.458***	0.515***	0.122***		1.55 + 0.30W + 0.39L^2^/R_50_ + 0.30S
W + L + S + E	0.822	0.474 (4.1%)	0.573***	0.355**	0.200**	0.062	–7.16 + 0.37W + 0.13L + 0.48S + 0.18E
W + L^2^/R_50_ + S + E	0.882	0.386 (3.4%)	0.464***	0.519***	0.125*	0.095	1.39 + 0.30W + 0.39L^2^/R_50_ + 0.30S + 0.28E

*aR*^*2*^ adjusted coefficient of determination, *E* ethnicity (Asian = 1, non-Asian = 0), *FFM* fat-free mass (kg), *L* standing height (cm), *L*^*2*^*/R*_*50*_ impedance index (cm^2^/ Ω), *RMSE* root mean squared error, *S* sex (M = 1, F = 0), *W* weight (kg).

*p < 0.05, **p < 0.01, ***p < 0.001 for statistically significant standardized regression coefficient from multivariable linear regression.

### Validation of fat-free mass equations

**Fig. 1:**
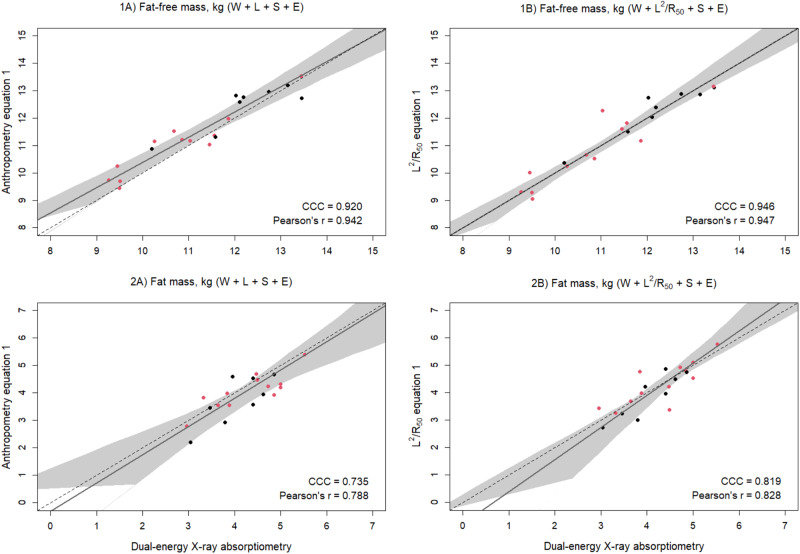
Scatterplots of predicted and reference fat-free mass (FFM) and fat mass (FM). Scatterplots of **1** fat-free mass (kg) and **2** fat mass (kg) of 3.5-year-old validation males (black) and females (red) (*n* = 20) measured with dual-energy X-ray absorptiometry and from prediction equations based on weight (W), sex (S), ethnicity (E), and **A** standing height (L) or **B** impedance index (L^2^/R_50_). Dashed lines are the lines of identity. Individual points below the line of identity indicate an underestimation, while those above are an overestimation. CCC is Lin’s concordance correlation coefficient and *r* is Pearson’s correlation coefficient.

When the final equations were validated, the MAPE for FFM was 3.8% for the anthropometry-based equation (W + L + S + E) and was reduced to 2.8% when using the impedance-based equation (W + L^2^/R_50_ + S + E). Similarly, concordance was improved for the impedance equation (Fig. 1).

**Fig. 2:**
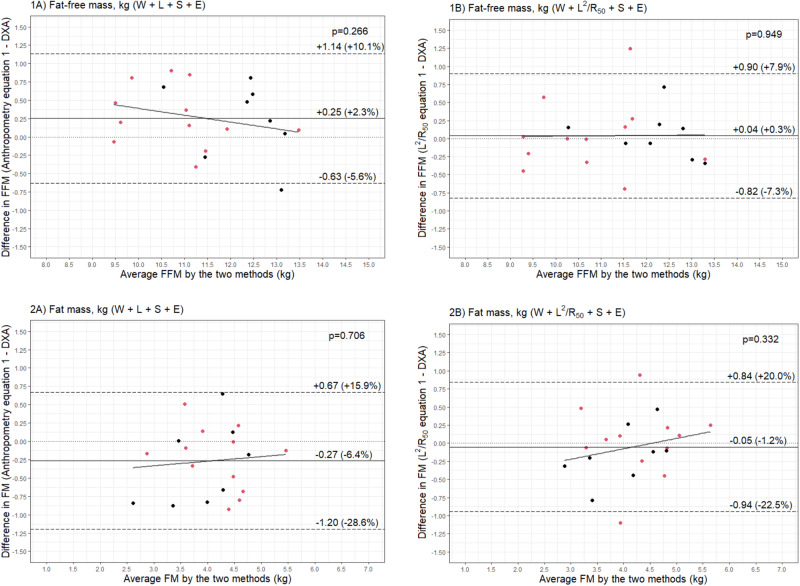
Bland-Altman plots of predicted and reference fat-free mass (FFM) and fat mass (FM). Bland–Altman plots comparing **1** fat-free mass (FFM) (kg) and **2** fat mass (FM) (kg) of 3.5-year-old validation males (black) and females (red) (*n* = 20) measured with dual-energy X-ray absorptiometry (DXA) and from prediction equations based on weight (W), sex (S), ethnicity (E), and **A** standing height (L) or **B** impedance index (L^2^/R_50_).

Bland–Altman analyses showed that the anthropometric equation could predict FFM with a bias of 250 g (2.3% of mean FFM), but with narrow limits of agreement that were equivalent to ±7.8% of mean FFM (Fig. 2). The impedance equation reduced the bias by approximately 200 g (40 g, 0.3% of mean FFM); however, limits of agreement were comparable at ±7.6% of mean FFM (Fig. 2). Both equations showed no statistically significant relationship between average FFM and the difference between measured and predicted FFM (Fig. 2).

The equations were then used to predict FM by subtracting the predicted FFM from the adjusted scale weight. The anthropometry equation predicted FM with a MAPE of 10.4%, while the impedance equation predicted FM with a lower error of 8.4%. Concordance and correlation were also improved with the impedance compared to the anthropometry equation (Fig. 1). Likewise, bias was reduced from –6.4% to –1.2% of mean FM, and limits of agreement narrowed (–0.27 kg [95% LOA: –1.20, 0.67] vs –0.05 kg [95% LOA: –0.94, 0.84]) (Fig. 2). There was no evidence of a proportional bias, with no statistically significant relationship between average FM and the difference between measured and predicted FM (Fig. 2).

Two one-sided tests of equivalence confirmed these findings. Only impedance-based estimates of FFM were considered equivalent to DXA estimates given equivalence bounds of ±250 g (–0.04 kg [90% CI: –0.21, 0.13], *p* = 0.022 vs +0.26 kg [90% CI: –0.43, –0.08], *p* = 0.52, respectively). However, neither impedance- nor anthropometry-based FM estimates were considered equivalent to DXA considering equivalence bounds of ±100 g (+0.05 kg [90% CI: –0.13, 0.23], *p* = 0.32 and +0.27 kg [90% CI: 0.08, 0.45], *p* = 0.93, respectively).

### Comparison to previously published equations

When the equations by Ejlerskov et al. [17] and Rush et al. [18] were validated in our cohort, the MAPE were large at 12.6% and 14.7%, respectively. Passing–Bablok regression scatterplots revealed that this error was due to the overestimation of FFM (Supplementary Fig. S2). While concordances were poor at 0.565 and 0.488, correlations were comparable to our equations (Supplementary Fig. S2). Bland–Altman analyses showed large biases of 1.4 kg (12.5%) and 1.7 kg (14.6%), with limits of agreement comparable to our equations at ±6.7% (Supplementary Fig. S3). There was no evidence to suggest that the relationship between measured and predicted FFM was influenced by body size (i.e., average FFM; Supplementary Fig. S3).

FM estimates were derived by subtracting FFM from scale weight (adjusted scale weight for Ejlerskov et al. [17]). MAPE were large for estimates of FM; they were improved when using the Ejlerskov equation compared to the Rush equation (38.5% vs 46.3%). Although correlations were comparable, concordance was improved for the Ejlerskov equation (Supplementary Fig. S2). Both equations underestimated FM, with the Rush equation doing so to a greater extent (–37.6% vs –45.0%). Nonetheless, both equations predicted FM with limits of agreement of approximately ±17% of mean FM and with no proportional bias (Supplementary Fig. S3).

### Mixture theory prediction

Each of the methods assessed predicted FFM with biases and limits of agreement that were larger than those observed when using the empirically derived equations (Table 3). Nonetheless, the default SFB7 coefficients, when combined with personalized Kb values, estimated FFM with a bias of less than 4% (–0.43 kg) and limits of agreement that were ±10.7% of mean FFM; however, a significant proportional bias was observed, with FFM being under- and overestimated among those with low and high levels of FFM, respectively (Table 3).

**Table 3:** Validation of mixture theory prediction of fat-free mass (FFM) against dual-energy X-ray absorptiometry (DXA) among a cohort of 3.5-year olds (*n* = 65).

	MAPE (%)	CCC	Bland–Altman analysis			
			Bias^a^	SD^a^	95% LOA	*p*
SFB7 default	7.00	0.777 (0.687, 0.843)	0.74 (6.5%)	0.67 (5.9%)	–0.57, 2.05	<0.001
SFB7 and personalized Kb	5.55	0.857 (0.790, 0.904)	–0.43 (–3.8%)	0.63 (5.5%)	–1.65, 0.79	<0.001
Moissl	7.44	0.740 (0.636, 0.818)	0.77 (6.7%)	0.74 (6.5%)	–0.68, 2.21	0.001
Xitron Hydra	18.23	0.404 (0.305, 0.494)	–2.05 (–18.0%)	0.61 (5.3%)	–3.25, –0.86	0.01
Xitron 4000B	12.55	0.567 (0.459, 0.658)	–1.41 (–12.3%)	0.55 (4.9%)	–2.49, –0.32	0.073
Xitron 4000B Ellis adjustments	13.55	0.541 (0.433, 0.633)	–1.52 (–13.4%)	0.59 (5.2%)	–2.69, –0.36	0.006

*MAPE* mean absolute percentage error, *CCC* Lin’s concordance correlation coefficient, *LOA* limits of agreement (±1.96 SD).

^a^Values are absolute (kg) and as a percentage of mean fat-free mass (11.4 kg) in parentheses.

## Discussion

This study developed and validated prediction equations for FFM using bioimpedance among a cohort of 3.5-year olds. Prediction equations incorporating bioimpedance performed better than simple equations based on weight, height, sex, and ethnicity. The final bioimpedance equation estimated FFM with a bias of 0.3% and limits of agreement of ±7.6% of mean FFM. The performance of our equations was similar to that of the previously published equations. However, when validated in our cohort, there were substantial biases for both published equations; FFM was overestimated by more than +12.5% of mean FFM. Each of the empirical equations assessed could more accurately estimate FFM at the individual level (i.e., narrower limits of agreement) than mixture theory prediction.

Rush et al. [18] developed the first bioimpedance prediction equation for FFM among young children using single-frequency BIA (ImpediMed BIM4) and DXA as the reference. The standard error of the estimate for the equation was 0.5 kg (equivalent to 4.6% of mean FFM), although they did not internally validate the equations. Nonetheless, the performance of their equation was markedly improved in comparison to previously published prediction equations [19, 21, 22], which each predicted FFM with biases of ≥1 kg and limits of agreement greater than ±1 kg when validated in their cohort [18]. Each of the published equations validated was developed among cohorts with wide age ranges, and varying associations between FFM and the impedance index according to age have previously been described [20].

When the Rush equation was externally validated by Ejlerskov et al. [17], the bias was low at 1.8% of mean FFM, and the limits of agreement were narrow (±7.2% of mean FFM). However, the bias was large for FM estimates (–12.3% of mean FM) and limits of agreement wider (±29.4% of mean FFM) [17]. Results were broadly comparable when Ejlerskov et al. [17] internally validated their bioimpedance equations, with limits of agreement of ±7.0% for FFM and ±28.8% for FM.

When we validated published equations in our cohort, substantial bias was evident for FFM (>12.5% of mean FFM); however, limits of agreement were narrow at approximately ±7%. The bias may be reflective of the different DXA devices used by the studies. Both Ejlerskov et al. [17] and Rush et al. [18] used a GE Lunar Prodigy as the reference. In contrast, we used a GE Lunar iDXA. Previously, we have reported substantial differences between body composition estimates from the Prodigy and the iDXA [27]. When examining limits of agreement, which reflect the degree of variation at the individual level, results were largely comparable. For example, when the NiPPeR equation was internally validated, FFM was predicted with limits of agreement that were ±7.6% of mean FFM. The Ejlerskov and Rush equations both predicted FFM with comparable limits of agreement of ±6.7% of mean FFM.

Previously, mixture theory coefficients appropriate for adults have been shown to be inapplicable for use in infancy [33, 39]; however, mixture theory prediction has seldom been evaluated in a cohort of healthy children. Ellis et al. [25] assessed the default Xitron 4000B method among a cohort of children (4–18 years, *n* = 347) and found that BIS estimates of TBW were inaccurate. Using a sub-set of their data (*n* = 116), they developed adjusted constants for this age group; although bias decreased, limits of agreement remained large at ±11–17% of mean TBW following recalibration. In our cohort, limits of agreement were narrower than that previously reported, though they were larger than was observed with the empirically derived equations. Notably, the default equation built into the SFB7 overestimated FFM by 0.74 kg, with limits of agreement that were ±11.5% of mean FFM. The inclusion of personalized Kb factors only marginally improved prediction.

Strengths of this study include the development of bioimpedance prediction equations in young children using the GE Lunar iDXA and the validation of published equations developed using the GE Lunar Prodigy. Our results confirm that the equations have similar performance, though differences exist in mean FFM estimates according to the DXA model used as the reference. We also evaluated whether the inclusion of additional covariates (ethnicity, gestational age, birthweight *z* score, time since the last meal, and time since the last bladder void) would improve the prediction of FFM. While we did not assess whether additional anthropometric measurements (e.g., skinfold thicknesses or circumferences) would improve prediction, previous reports showed minimal improvements [17, 33]. Further limitations include a reduced sample size (after setting aside a third of participants for validation) which may have impacted our ability to assess the contribution of the aforementioned covariates. However, the final prediction equation explained 88% of the variance in FFM, with weight and the impedance index explaining the majority of the variance (*β*: 0.464 and 0.519, respectively). In addition, the study is limited by the use of DXA rather than a multicomponent model gold standard as the reference, which may have led to the overestimation of FM [40].

In summary, our prediction equation based on weight, the impedance index, and sex estimated FFM and FM with biases of less than 2%. Limits of agreement were acceptably narrow at less than ±8% of mean FFM, but wider for FM. Body composition estimates were improved when using the impedance compared to the anthropometry-based equations. Although previously published equations had similar individual-level performance, substantial bias was evident, highlighting the importance of considering the reference standard used, particularly when longitudinal analyses are being conducted. Our equations provide an easy method for estimating body composition in preschool children; however, further external validation of the equations is recommended.

## Supplementary information

Supplementary file revised

## Data Availability

Data described in the manuscript, code book, and analytic code will not be made available because the participants did not consent to open access data sharing and this is an ongoing longitudinal study in which there will be further future analyses conducted.

## Code availability

Codes for analyses are available on reasonable request from the corresponding author.
