# Supplementary material for: Prediction of fat-free mass in young children using bioelectrical impedance spectroscopy
Source: Eur J Clin Nutr. 2023 Jul 31;78(10):872–9. doi: 10.1038/s41430-023-01317-4 (PMC7616480; doi:10.1038/s41430-023-01317-4)
Supplement: Supplementary file 1 — Supplementary file revised [file 41430_2023_1317_MOESM1_ESM.docx]

**Prediction of fat-free mass in young children using bioelectrical impedance spectroscopy**

Jaz Lyons-Reid^1^, Leigh C. Ward^2^, José G. B. Derraik^1,3,4,5^, Mya Thway-Tint^6,7^, Cathriona R. Monnard^8^, Jose M. Ramos Nieves^8^, Benjamin B. Albert^1^, Timothy Kenealy^1,9^, Keith M. Godfrey^10,11^, Shiao-Yng Chan^6,12^, Wayne S. Cutfield^*1,13^

^1^Liggins Institute, The University of Auckland, Auckland, New Zealand

^2^School of Chemistry and Molecular Biosciences, The University of Queensland, Brisbane, Australia

^3^Department of Paediatrics: Child and Youth Health, School of Medicine, Faculty of Medical and Health Sciences, University of Auckland, Auckland, New Zealand

^4^Environmental-Occupational Health Sciences and Non-communicable Diseases Research Group, Research Institute for Health Sciences, Chiang Mai University, Chiang Mai, Thailand

^5^Department of Women’s and Children’s Health, Uppsala University, Uppsala, Sweden

^6^Singapore Institute for Clinical Sciences, Agency for Science, Technology and Research (A*STAR), Singapore

^7^Human Potential Translational Research Programme, Yong Loo Lin School of Medicine, National University of Singapore, Singapore

^8^Nestlé Institute of Health Sciences, Nestlé Research, Société des Produits Nestlé S.A., Lausanne, Switzerland

^9^Department of Medicine and Department of General Practice and Primary Health Care, The University of Auckland, Auckland, New Zealand

^10^MRC Lifecourse Epidemiology Centre, University of Southampton, Southampton, United Kingdom

^11^NIHR Southampton Biomedical Research Centre, University of Southampton and University Hospital Southampton NHS Foundation Trust, Southampton, United Kingdom

^12^Department of Obstetrics & Gynaecology, National University of Singapore, Singapore

^13^A Better Start – National Science Challenge, The University of Auckland, Auckland, New Zealand

**Corresponding author:** Professor Wayne Cutfield; mailing address Private Bag 92019, Auckland 1142, NZ; telephone +64 9 923 4476; email w.cutfield@auckland.ac.nz

**Supplementary Table 1** Inclusion and exclusion criteria of the NiPPeR study (adapted from Godfrey et al. Trials. 2017; 18:131).

| Inclusion criteria | - Aged 18–38 years - Living in Southampton, Singapore or Auckland - In Southampton and Auckland, planning to have future maternity care in Southampton and Auckland, respectively - In Singapore, willing to deliver at the National University Hospital - Women planning to conceive within 6 months (but conception up to 12 months after phenotyping will still be included) - In Singapore only women of Chinese, Malay and Indian ethnicity, or of mixed Chinese/Malay/Indian ethnicity will be included - Able to provide written, informed consent |
| --- | --- |
| Exclusion criteria | - Pregnant or lactating at recruitment (women who are currently breastfeeding will be excluded, but no washout period from the end of breastfeeding will be required before study start) - Assisted fertility apart from those taking clomiphene or letrozole alone - Women with pre-existing type-1 or type-2 diabetes (fasting plasma glucose concentration ≥7.0 mmol/L or post OGTT 2-h plasma glucose concentration ≥11.1 mmol/L) - Oral or implanted contraception currently or in the last month, or with an intrauterine contraceptive device in situ - Metformin or systemic steroids currently or in the last month - Anticonvulsant medication currently or in the last month - Treatment for HIV, Hepatitis B or C currently or in the last month - Known serious food allergy |

**
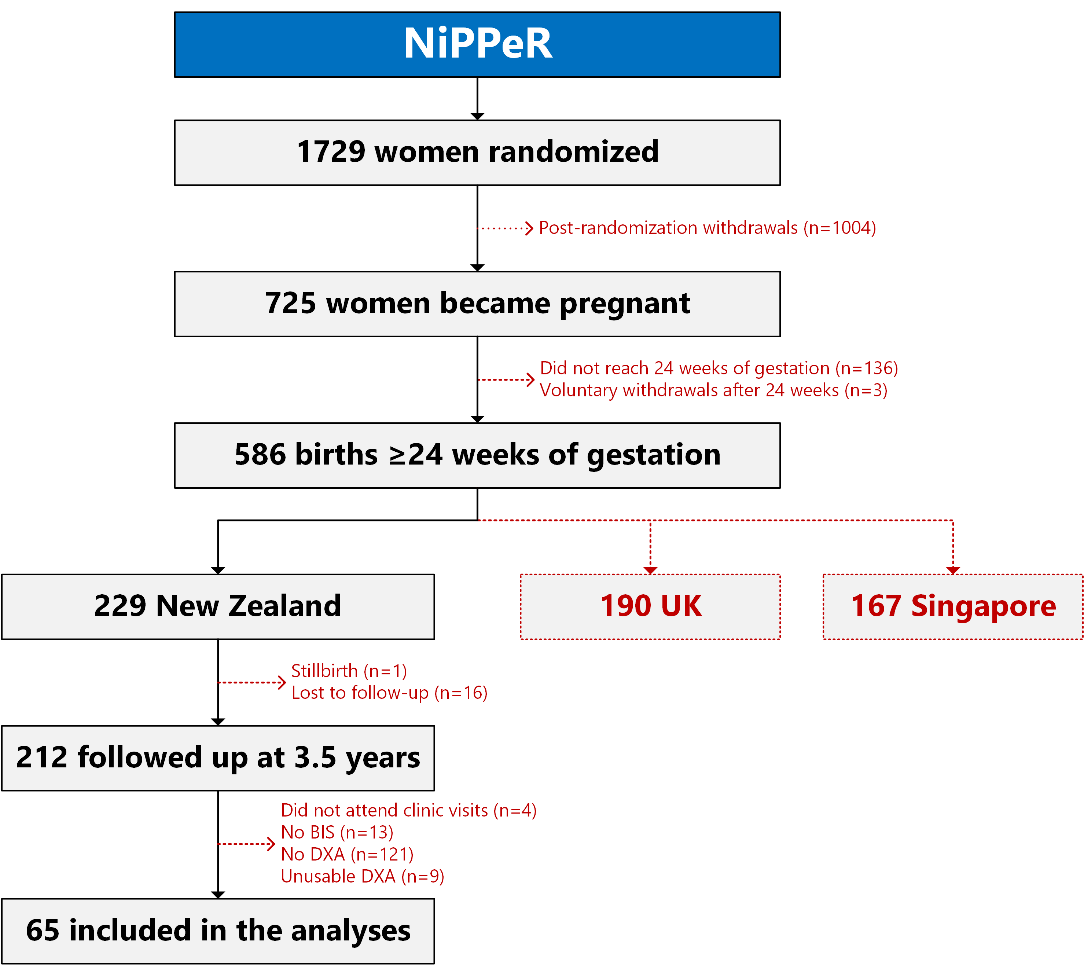
**

**Supplementary Figure 1** CONSORT diagram of participants enrolled in the NiPPeR trial 3.5-years after delivery.

**Supplementary Table 2** Characteristics of the derivation and validation populations.

|  | Derivation  (n=45) | Validation  (n=20) |
| --- | --- | --- |
| Males (%) | 17 (37.8%) | 8 (40.0%) |
| Gestational age at birth (weeks) | 39.5 ± 1.6 | 39.7 ± 1.6 |
| Birthweight SDS^+^ | 0.35 ± 1.02 | 0.28 ± 1.02 |
| Age at visit (days) | 1227 ± 50 | 1244 ± 90 |
| Scale weight (kg) | 15.6 ± 1.8 | 15.3 ± 1.7 |
| Weight SDS^&^ | 0.36 ± 0.88 | 0.20 ± 0.89 |
| Standing height (cm) | 99.4 ± 3.3 | 98.8 ± 4.1 |
| Height SDS^&^ | 0.36 ± 0.82 | 0.20 ± 1.03 |
| BMI (kg/m^2^) | 15.7 ± 1.2 | 15.6 ± 1.0 |
| BMI SDS^&^ | 0.19 ± 0.87 | 0.10 ± 0.77 |
| Fat-free mass^^^ (kg) | 11.5 ± 1.2 | 11.3 ± 1.3 |
| Fat mass^^^ (kg) | 4.4 ± 1.0 | 4.2 ± 0.7 |
| Fat mass^^^ (%) | 27.4 ± 4.0 | 27.1 ± 3.5 |
| Lean mass^^^ (kg) | 11.0 ± 1.1 | 10.8 ± 1.3 |
| Bone mineral content^^^ (g) | 530 ± 60 | 524 ± 63 |
| Resistance at 0 kHz (Ω) | 810 ± 64 | 812 ± 96 |
| Resistance at ∞ kHz (Ω) | 612 ± 54 | 623 ± 82 |
| Impedance at Fc^$^ (Ω) | 714 ± 59 | 720 ± 89 |
| Resistance at 50 kHz (Ω) | 741 ± 60 | 747 ± 89 |
| Ethnicity | | |
| – White Caucasian | 33 (73.3%) | 13 (65.0%) |
| – Chinese | 6 (13.3%) | 3 (15.0%) |
| – South Asian | 3 (6.7%) | 2 (10.0%) |
| – Other | 9 (6.7%) | 2 (10.0%) |
| Randomisation group | | |
| – Intervention | 22 (48.9%) | 10 (50.0%) |
| – Control | 23 (51.1%) | 10 (50.0%) |
| Data are means ± SD for continuous variables and n (%) for categorical variables. ^+^INTERGROWTH-21^st^ birthweight standard deviation scores (SDS). ^&^World Health Organization age- and sex-standardised deviation scores (SDS). ^^^Whole-body body composition estimates from DXA. ^$^Impedance at the characteristic frequency (Fc). | | |

**
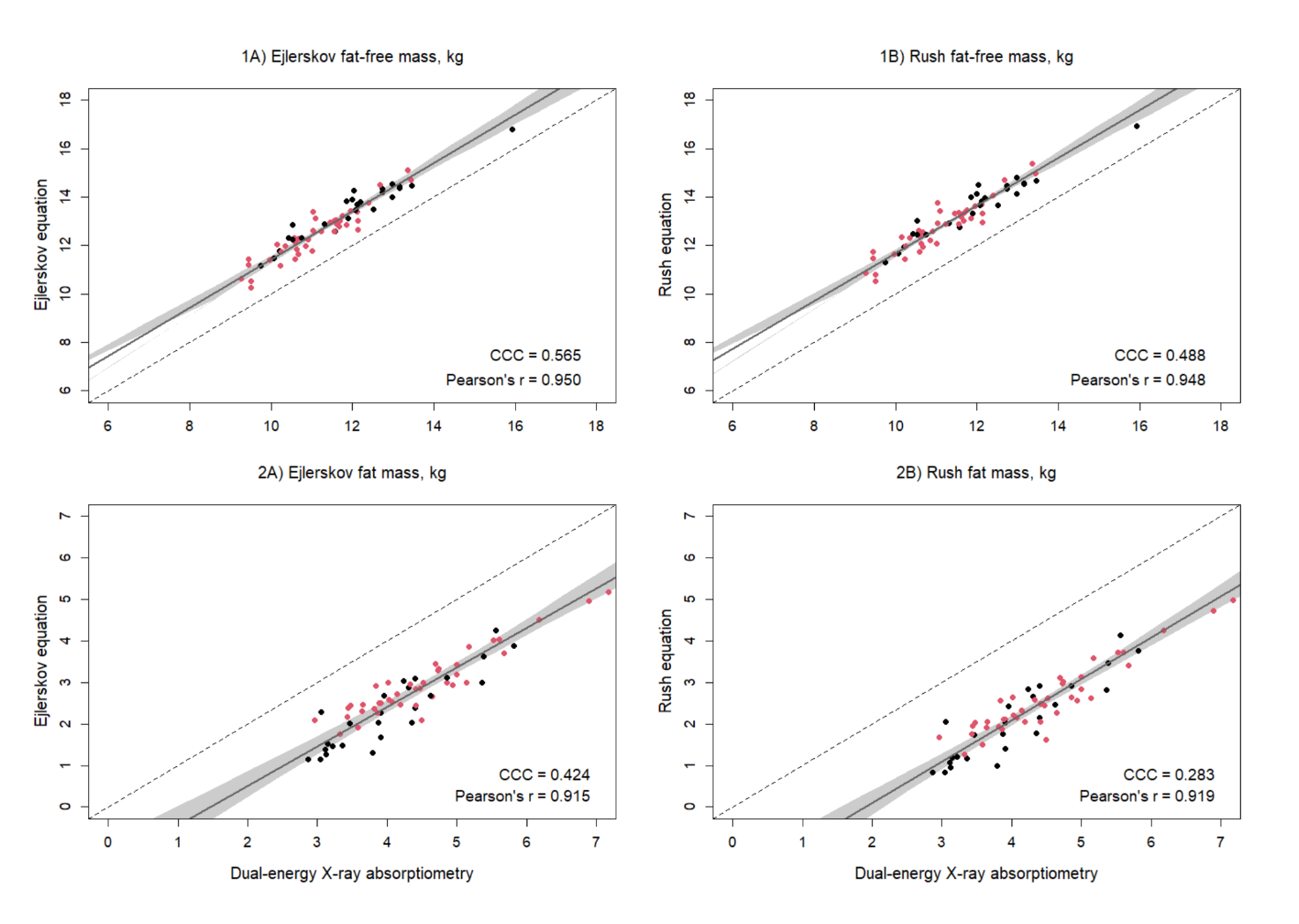
Supplementary Figure 2** Scatterplots of 1) fat-free mass (kg) and 2) fat mass (kg) of 3.5-year-old males (black) and females (red) (n=65) measured with dual-energy x-ray absorptiometry and from prediction equations from A) Ejlerskov and B) Rush, based on weight (W), the impedance index (L2/R50), standing height (Ht), and sex (S). Dashed lines are the lines of identity. Individual points below the line of identity indicate an underestimation, while those above are an overestimation. CCC is Lin’s concordance correlation coefficient and r is Pearson’s correlation coefficient.

**
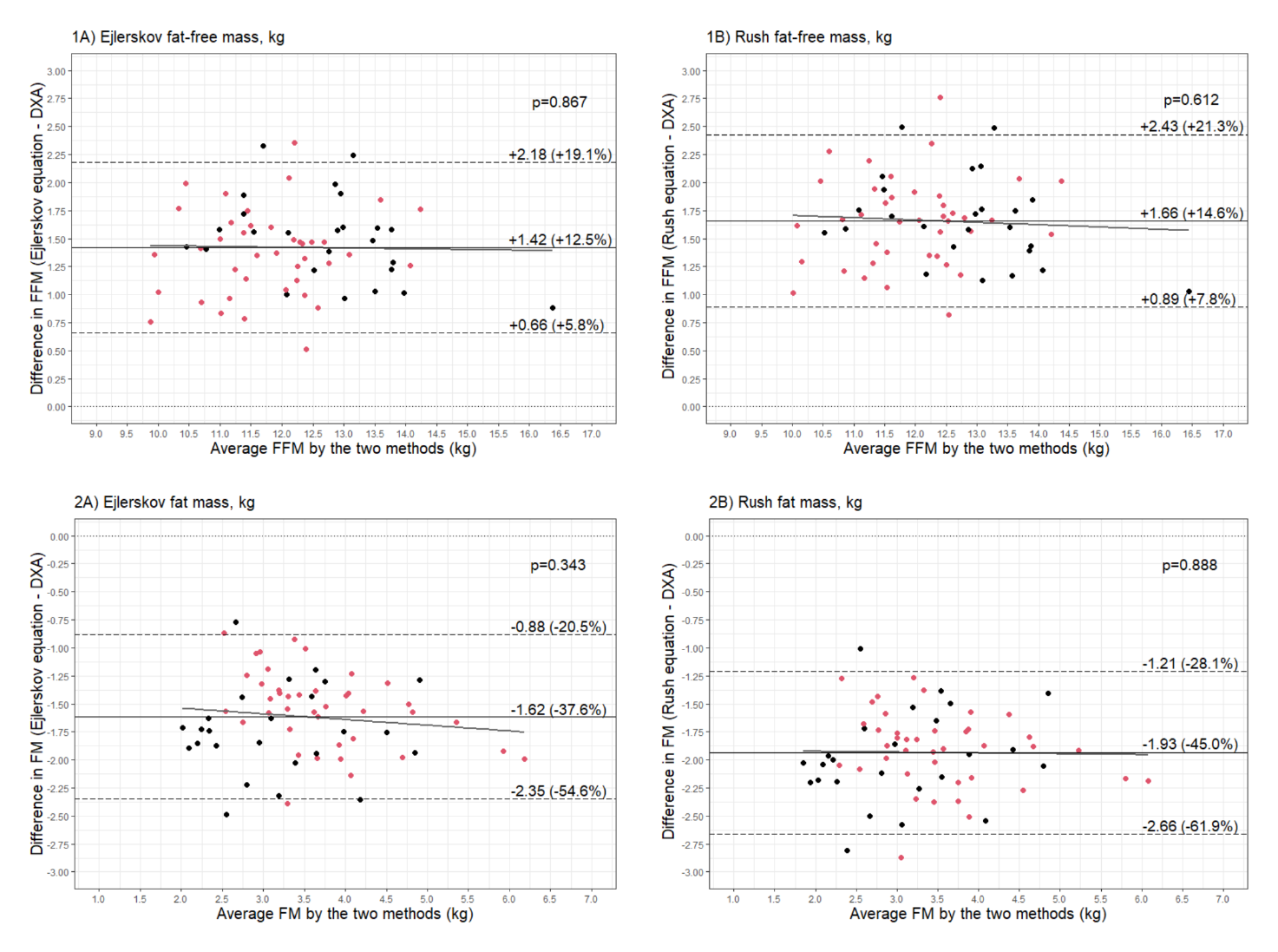
**

**Supplementary Figure 3** Bland–Altman plots comparing 1) fat-free mass (FFM) (kg) and 2) fat mass (FM) (kg) of 3.5-year-old males (black) and females (red) (n=65) measured with dual-energy X-ray absorptiometry (DXA) and from prediction equations from A) Ejlerskov and B) Rush, based on weight (W), the impedance index (L2/R50), standing height (Ht), and sex (S).
